# The combination of temozolomide-irinotecan regresses a doxorubicin-resistant patient-derived orthotopic xenograft (PDOX) nude-mouse model of recurrent Ewing’s sarcoma with a FUS-ERG fusion and *CDKN2A* deletion: Direction for third-line patient therapy

**DOI:** 10.18632/oncotarget.20789

**Published:** 2017-09-08

**Authors:** Kentaro Miyake, Takashi Murakami, Tasuku Kiyuna, Kentaro Igarashi, Kei Kawaguchi, Masuyo Miyake, Yunfeng Li, Scott D. Nelson, Sarah M. Dry, Michael Bouvet, Irmina A. Elliott, Tara A. Russell, Arun S. Singh, Mark A. Eckardt, Yukihiko Hiroshima, Masashi Momiyama, Ryusei Matsuyama, Takashi Chishima, Itaru Endo, Fritz C. Eilber, Robert M. Hoffman

**Affiliations:** ^1^ AntiCancer Inc., San Diego, CA, USA; ^2^ Department of Surgery, University of California, San Diego, CA, USA; ^3^ Department of Gastroenterological Surgery, Yokohama City University Graduate School of Medicine, Yokohama, Japan; ^4^ Division of Hematology-Oncology, University of California, Los Angeles, CA, USA; ^5^ Department of Pathology, University of California, Los Angeles, CA, USA; ^6^ Division of Surgical Oncology, University of California, Los Angeles, CA, USA; ^7^ Department of Surgery, Yale School of Medicine, New Haven, CT, USA

**Keywords:** Ewing’s sarcoma, patient-derived orthotopic xenograft, irinotecan, temozolomide, third-line chemotherapy

## Abstract

The aim of the present study was to determine the usefulness of a patient-derived orthotopic xenograft (PDOX) nude-mouse model of a doxorubicin-resistant metastatic Ewing’s sarcoma, with a unique combination of a FUS-ERG fusion and *CDKN2A* deletion, to identify effective drugs for third-line chemotherapy of the patient. Our previous study showed that cyclin-dependent kinase 4/6 (CDK4/6) and insulin-like growth factor-1 receptor (IGF-1R) inhibitors were effective on the Ewing’s sarcoma PDOX, but not doxorubicin, similar to the patient’s resistance to doxorubicin. The results of the previous PDOX study were successfully used for second-line therapy of the patiend. In the present study, the PDOX mice established with the Ewing’s sarcoma in the right chest wall were randomized into 5 groups when the tumor volume reached 60 mm^3^: untreated control; gemcitabine combined with docetaxel (intraperitoneal [i.p.] injection, weekly, for 2 weeks); irinotecan combined with temozolomide (irinotecan: i.p. injection; temozolomide: oral administration, daily, for 2 weeks); pazopanib (oral administration, daily, for 2 weeks); yondelis (intravenous injection, weekly, for 2 weeks). All mice were sacrificed on day 15. Body weight and tumor volume were assessed 2 times per week. Tumor weight was measured after sacrifice. Irinotecan combined with temozolomide was the most effective regimen compared to the untreated control group (p=0.022). Gemcitabine combined with docetaxel was also effective (p=0.026). Pazopanib and yondelis did not have significant efficacy compared to the untreated control (p=0.130, p=0.818). These results could be obtained within two months after the physician’s request and were used for third-line therapy of the patient.

## INTRODUCTION

Ewing’s sarcoma (ES) is a rare and aggressive disease that mostly effects children and teenagers [1, 2]. The development of multi-agent chemotherapy has improved outcome [3–6], but is not effective for ES patients with metastasis [7]. Furthermore, the heterogeneity of ES makes the treatment decisions much more complicated [8, 9].

We previously established a patient-derived orthotopic xenograft (PDOX) models of a rare case of ES with both a *FUG-ERG* fusion [10, 11] and a loss of the *CDKN2A*. Previously, we reported that a CDK4/6 inhibitor and insulin-like growth factor-1 receptor (IGF-1R) inhibitor were effective in the ES PDOX model [12]. The PDOX tumor was resistant to doxorubicin (DOX) as was the patient [12]. ES recurred in the bone marrow of the patient 19 months after primary tumor resection and DOX treatment. Based on the PDOX results, an IGF-1R inhibitor was used successfully as a second line therapy for the bone marrow recurrence in the patient. The patient’s physician requested a subsequent PDOX test and the results of the present study were used for treatment of remaining organ metastasis.

## RESULTS AND DISCUSION

**Figure 1:**
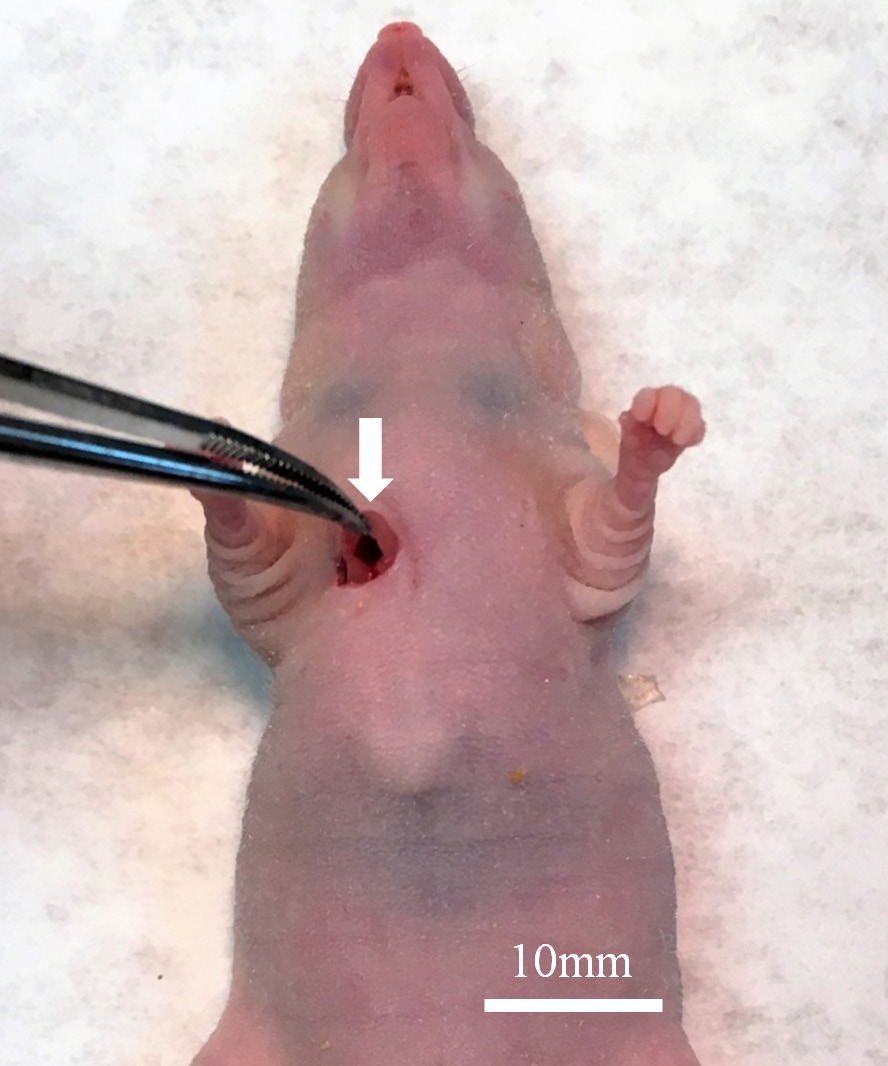
Surgical orthotopic implantation of Ewing’s sarcoma (ES) tumor A space between the pectoral muscle (white arrow) and intercostal muscle was made in the right chest wall of nude mice for orthotopic implantation of the ES tumor.

**Figure 2:**
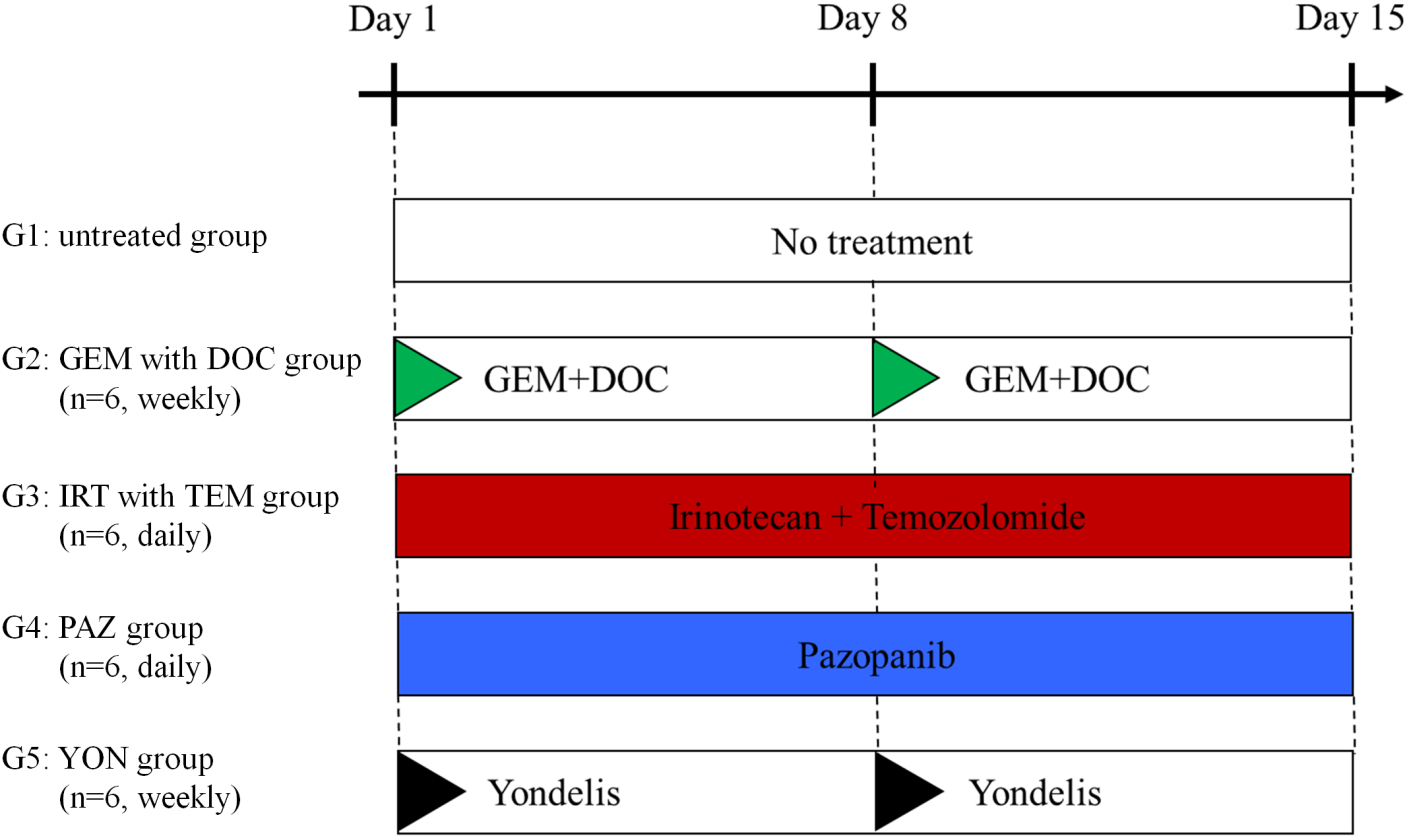
Treatment protocol for ES PDOX G1: untreated control; G2: combination treatment with GEM + DOC (GEM: intraperitoneal [i.p.], 100 mg/kg, weekly, 2 weeks, DOC: i.p., 20 mg/kg, weekly, 2 weeks); G3: combination treatment with IRT + TEM (IRT: i.p., 4 mg/kg, daily, 2 weeks, TEM: oral [p.o.], 25 mg/kg, daily, 2 weeks); G4: PAZ (p.o., 100 mg/kg, daily, 2 weeks); G5: YON (intravenous [i.v.], 0.15 mg/kg, weekly, 2 weeks). Each group consisted of n=6 mice. All mice were sacrificed on day 15. GEM=gemcitabine; DOC=docetaxel; IRT=irinotecan; TEM=temozolomide.

**Figure 3:**
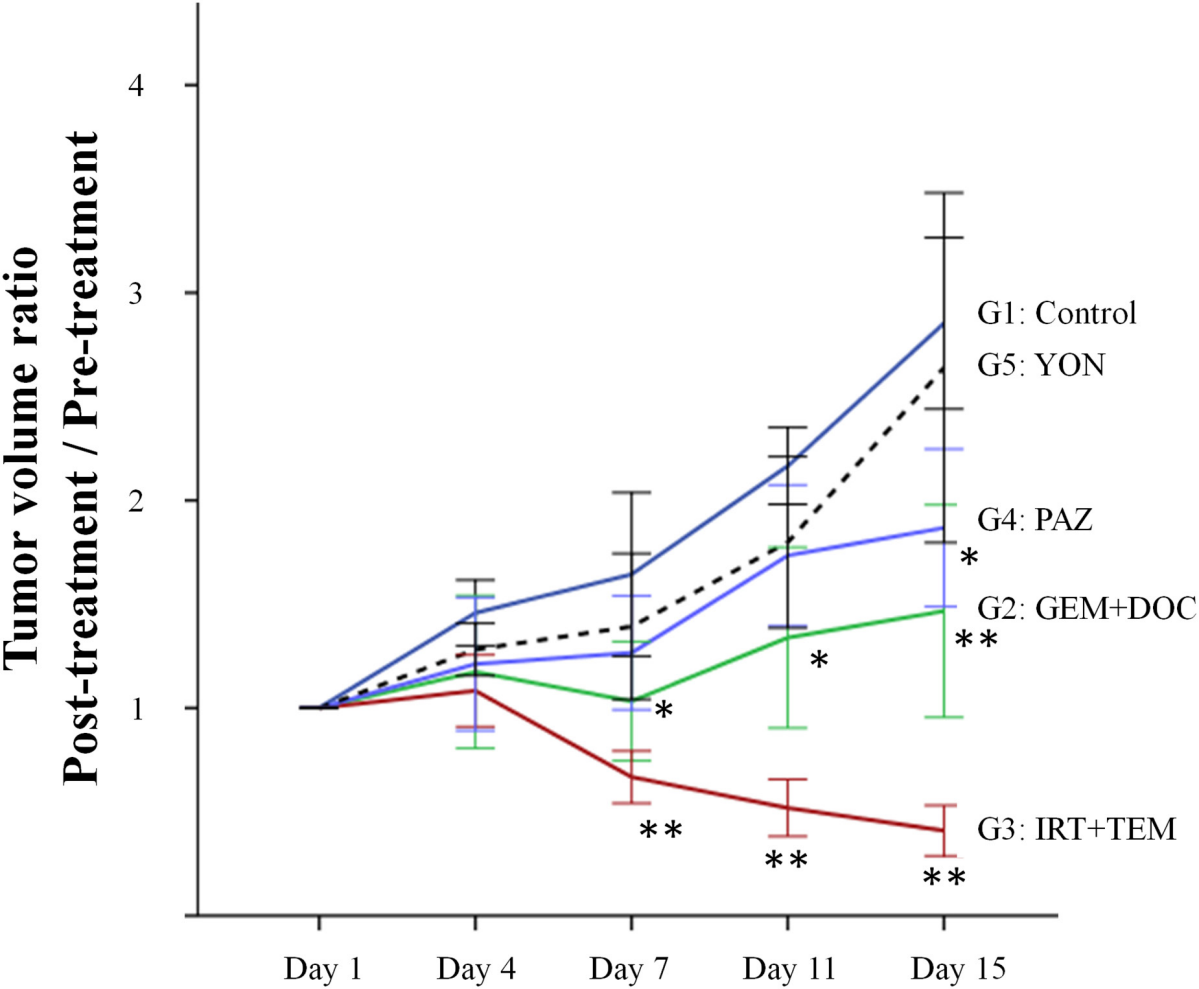
Time course of tumor volume ratio in treated mice compared to untreated control Line graphs indicate tumor volume ratio (post-treatment volume / pre-treatment volume) on each tumor-measurement day. IRT combined with TEM regressed tumor growth significantly compared to the untreated control group on day 7, 11 and 15 (p<0.001). There was also a significant difference between the untreated control group and the mice treated with the combination of GEM with DOC group on day 7, 11, and 15 (p=0.004, p=0.002, and p=0.001), respectively. PAZ suppressed the tumor growth significantly on day 15 (p=0.001). ^*^P<0.01, ^**^P<0.001 compared to untreated group. Error bars: ± 1 SD.

The site of implantation of the ES in the nude mice is shown in Figure 1. The treatment schema is illustrated in Figure 2. The time-course change of the tumor volume ratio is shown in Figure 3. The combination of irinotecan (IRT) with temozolomide (TEM) was most effective, and showed significant tumor regression compared to the untreated control group on day 15 (p<0.001). There was also a significant difference between the untreated control group and the mice treated with the combination of gemcitabine (GEM) with docetaxel (DOC) on day 15 (p=0.001). Pazopanib (PAZ) suppressed the tumor growth significantly on day 15 (p=0.001). Yondelis (YON) did not significantly suppress tumor growth (p=0.342). Final relative tumor volume ratios on day 15 compared to day 1 were as follows: untreated control group (G1) (3.13 ± 0.5); combination with GEM with DOC (G2) (1.47 ± 0.47); combination with IRT with TEM (G3) (0.41 ± 0.11); PAZ (G4) (1.87 ± 0.35); YON (G5) (2.64 ± 0.35). The 0.41 relative tumor volume of the IRT-TEM group indicates tumor regression, which is important for clinical translation of the efficacy of IRT-TEM for the patient [13].

**Figure 4:**
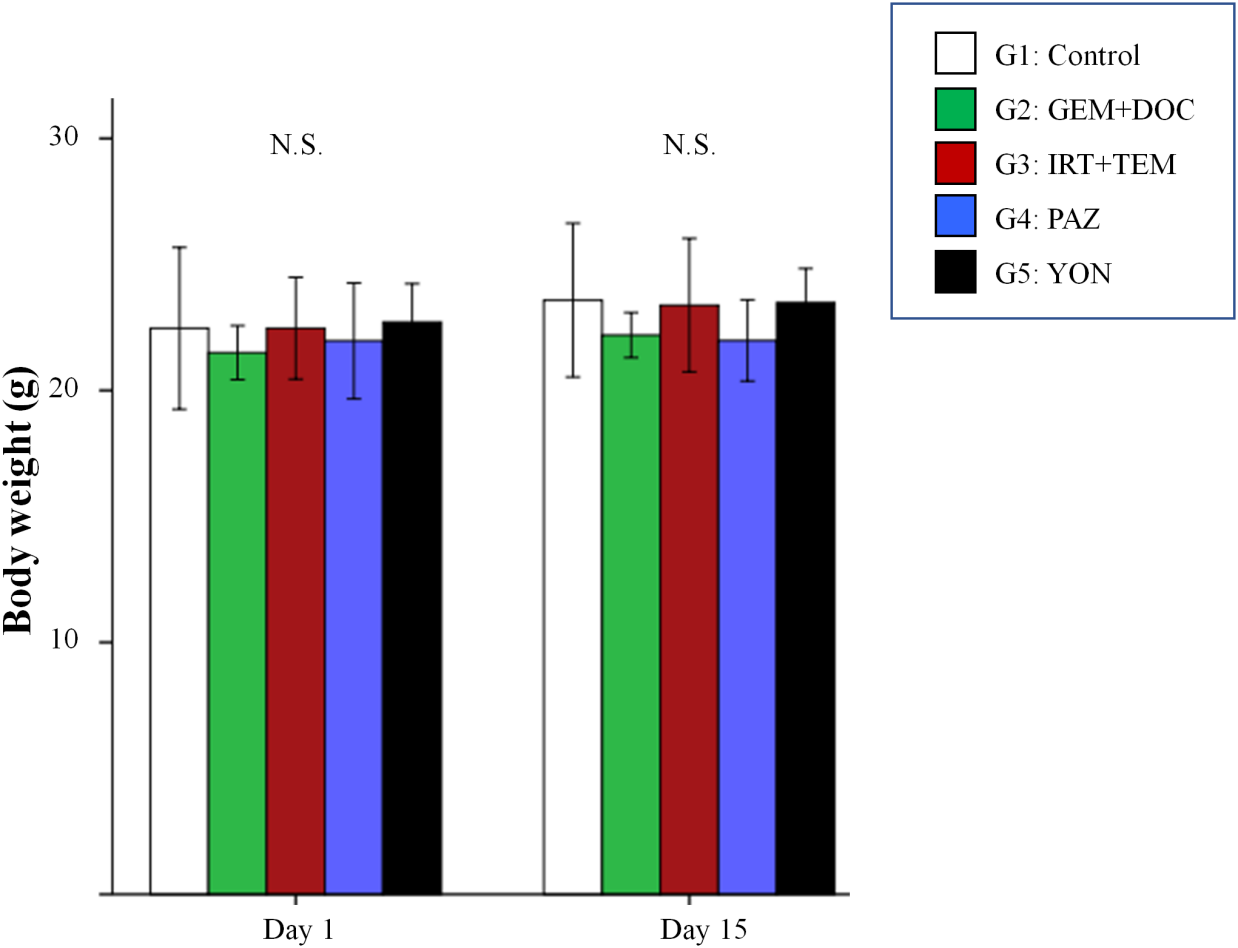
Body weight of treated versus untreated mice Bar graphs indicate body weight in each group on day 1 and 15. No significant difference was observed between any group. Error bars: ± 1 SD.

There was no significant difference in body weight on day 1 and day 15 between the 5 groups (Figure 4) suggesting there was no acute toxicity due to any of the treatments.

**Figure 5:**
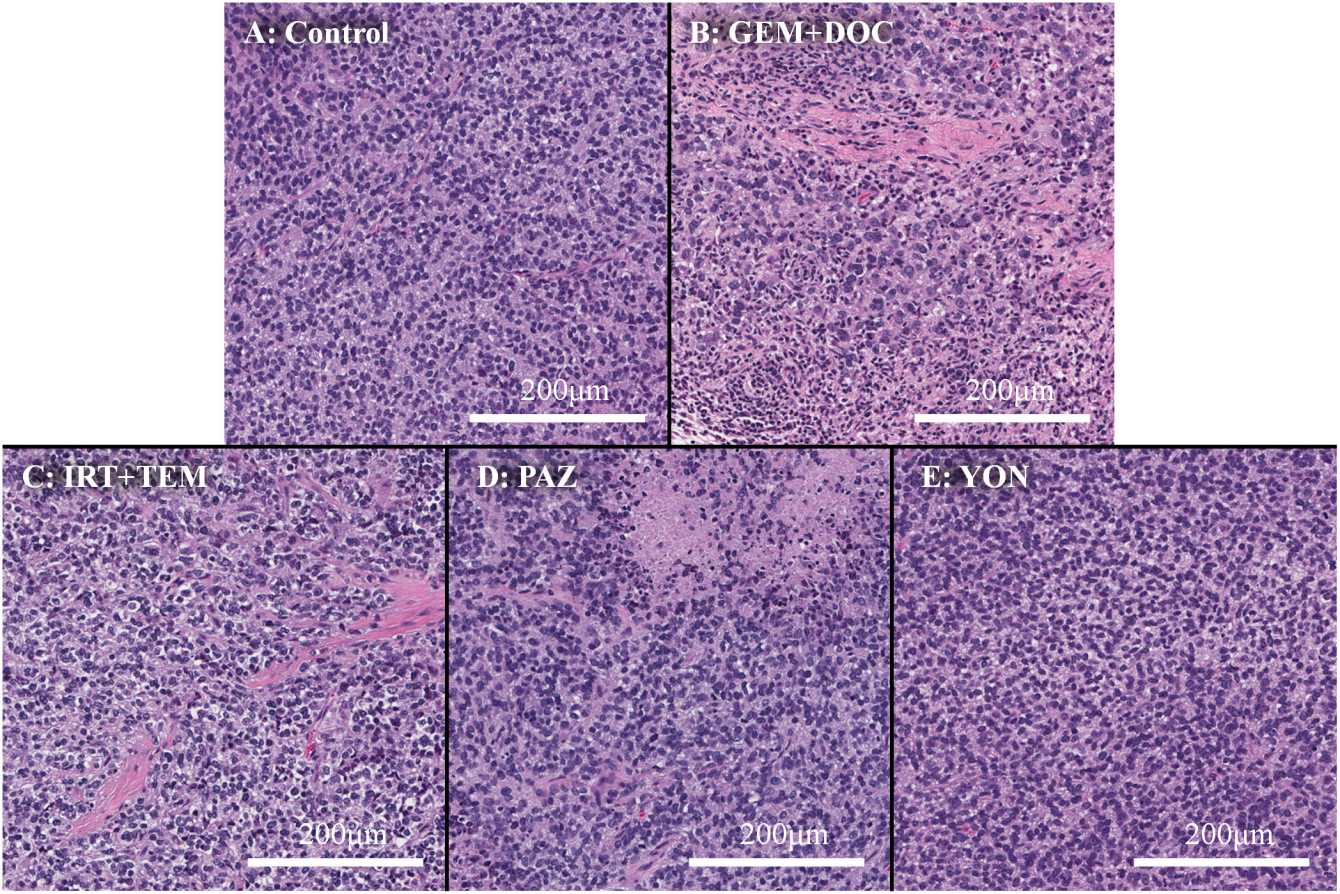
Histopathology **(A)** Hematoxylin and eosin (H&E) staining of the untreated PDOX tumor. **(B)** H&E staining of a tumor treated with the combination of GEM and DOC. **(C)** H&E staining of a tumor treated with the combination of IRT and TEM. **(D)** H&E staining of a tumor treated with PAZ. **(E)** H&E staining a tumor treated with YON. Necrosis was observed in all treatment groups other than YON.

Hematoxylin and eosin (H&E)-staining of tumor tissue sections showed necrosis due to treatment with all drugs except YON (Figure 5). The previously-established ES PDOX model had similar histological findings compared to the original patient’s tumor [12].

Toward the goal of precision personalized oncology, our laboratory pioneered the patient-derived orthotopic xenograft (PDOX) nude mouse model with the technique of surgical orthotopic implantation (SOI), including pancreatic [14–18], breast [19], ovarian [20], lung [21], cervical [22, 23], colon [24–26], and stomach cancer [27], sarcoma [12, 28–37] and melanoma [38–42].

The heterogeneity of ES makes individualized therapy particularly pertinent for this disease and the PDOX model can play an important role in achieving this goal.

Based on the efficacy of an IGF-1R inhibitor in the ES PDOX model [12], a compassionate-use investigational new drug (IND) approval was obtained from the U.S. Food and Drug Administration (FDA) for second-line treatment of the ES patient with an IGF-1R inhibitor, which resulted in clearance of the ES from the patient’s bone marrow (Eilber, F.C, et al., unpublished results), thereby allowing subsequent cytotoxic chemotherapy to be administered for third line therapy. We obtained the present results within only 2 months from receiving the physician’s request using the ES PDOX model, in time for third-line therapy guidance. These results thereby demonstrate an important clinical use of the PDOX model.

The ES PDOX was also shown to be sensitive to experimental therapeutics including recombinant methioninase [43] and *Salmonella typhimurium* A1-R [34].

Previously-developed concepts and strategies of highly-selective tumor targeting can take advantage of molecular targeting of tumors, including tissue-selective therapy which focuses on unique differences between normal and tumor tissues [44–49].

## CONCLUSIONS

An effective drug combination was identified using the PDOX model for recurrent Ewing’s sarcoma within a time frame to design a treatment strategy for third line therapy of the patient, demonstrating the power of the PDOX model for individualized therapy.

## MATERIALS AND METHODS

### Mice

Athymic *nu/nu* female nude mice (AntiCancer Inc., San Diego, CA, USA), 4–6 weeks old, were used in this study. Animals were housed in a barrier facility on a high efficiency particulate arrestance (HEPA)-filtered rack under standard conditions of 12-hour light/dark cycles. The animals were fed an autoclaved laboratory rodent diet [12]. All animal studies were conducted with an AntiCancer Institutional Animal Care and Use Committee (IACUC)-protocol specifically approved for this study and in accordance with the principals and procedures outlined in the National Institutes of Health Guide for the Care and Use of Animals under Assurance Number A3873-1. In order to minimize any suffering of the animals the use of anesthesia and analgesics were used for all surgical experiments. Animals were anesthetized by subcutaneous injection of a 0.02 ml solution of 20 mg/kg ketamine, 15.2 mg/kg xylazine, and 0.48 mg/kg acepromazine maleate. The response of animals during surgery was monitored to ensure adequate depth of anesthesia. The animals were observed on a daily basis and humanely sacrificed by CO_2_ inhalation when they met the following humane endpoint criteria: severe tumor burden (more than 20 mm in diameter), prostration, significant body weight loss, difficulty breathing, rotational motion and body temperature drop.

### Previous establishment of the ES PDOX model

The ES tumor recurred in the right chest wall of the patient [12]. The patient received neoadjuvant multidrug chemotherapy using doxorubicin, vincristine, and cyclophosphamide. Then, curative intent surgery was performed in the Department of Surgery, University of California, Los Angeles, USA (UCLA) and a portion of the tumor was previously used for establishment of a PDOX model in the right chest wall of nude mice [12]. Informed consent was previously obtained from the patient, and this study was approved by the Institutional Review Board of UCLA. Fresh tumor was brought to AntiCancer Inc. from the UCLA Hospital [12]. The ES PDOX was established by implantation between the pectoral muscle and intercostal muscle in the right chest wall of nude mice [12] (Figure 1).

### Treatment protocol for the ES PDOX model

The PDOX mice were randomized into 5 groups before tumor volume reached 60 mm^3^: G1: untreated control; G2: gemcitabine (GEM) combined with docetaxel (DOC) (GEM: i.p., 100 mg/kg, weekly, 2 weeks, DOC: i.p., 20 mg/kg, weekly, 2 weeks); G3: irinotecan (IRT) with temozolomide (TEM) (IRT: i.p., 4 mg/kg, daily, 2 weeks, TEM: p.o., 25 mg/kg, daily, 2 weeks); G4: pazopanib (PAZ) (p.o., 100 mg/kg, daily, 2 weeks); G5: yondelis (YON) (i.v., 0.15 mg/kg, weekly, 2 weeks) (Figure 2). Drug dosages were determined using previous reports (13-16). Tumor size and body weight were measured 2 times a week. Tumor volume was calculated with the following formula: tumor volume (mm^3^) = length (mm) x width (mm) x width (mm) x ½ [12]. After 2 weeks, all mice were sacrificed.

### Histological examination

Fresh tumor samples were fixed in 10% formalin and embedded in paraffin before sectioning and staining. Tissue sections (5 μm) were deparaffinized in xylene and rehydrated in an ethanol series. Hematoxylin and eosin (H&E) staining was performed according to standard protocols. Histological examination was performed with a BHS System Microscope (Olympus Corporation,Tokyo, Japan). Images were acquired with INFINITY ANALYZE software (Lumenera Corporation, Ottawa, Canada) [12].

### Statistical analysis

All statistical analyses were performed with the Statistical Package for the Social Sciences for Windows software version 22.0 (IBM Corp., Armonk, NY, USA). Significant differences for continuous variables were determined using the Mann-Whitney U test. Line graphs show the median and error bars indicate ± standard deviation. A probability value of P ≤ 0.05 was defined as statistically-significant [12].
